# Health Care Professionals’ Estimation of Home Spirometry Use Among People With Cystic Fibrosis: Cross-Sectional Study

**DOI:** 10.2196/93545

**Published:** 2026-08-26

**Authors:** Aqeem Azam, Andrew M Jones, Rowland J Bright-Thomas, Alex Horsley, Peter J Barry

**Affiliations:** 1 Manchester Adult Cystic Fibrosis Centre, Manchester University NHS Foundation Trust, Southmoor Road, Wythenshawe, Manchester, England, M23 9LT, United Kingdom, 44 161 291 5120; 2 Division of Immunology, Immunity to Infection and Respiratory Medicine, University of Manchester, Manchester, England, United Kingdom

**Keywords:** spirometry, cystic fibrosis, telehealth, telemonitoring, home spirometry, remote monitoring

## Abstract

**Background:**

Home spirometry has been widely adopted in the delivery of cystic fibrosis (CF) care. While existing literature largely supports its feasibility and positive outcomes, behavior around home disease monitoring remains poorly understood. Inaccurate assumptions about home spirometry behavior may affect resource prioritization and influence clinical decisions and follow-up.

**Objective:**

This study aimed to evaluate health care professionals’ (HCPs’) ability to estimate home spirometry use among people with CF and compare these estimates with actual recorded data.

**Methods:**

Home spirometry data from 48 people with CF attending a large adult CF center in the United Kingdom were obtained from the NuvoAir platform for the period from January to December 2024. The home spirometry data were sampled to represent 3 predefined use categories: infrequent, expected, and highly frequent users. A paper-based survey was distributed to HCPs with experience using home spirometry within the CF service, including physicians, CF specialist nurses, and physiotherapists, with survey dissemination and data collection completed in January 2025. Participants rated their familiarity with each person with CF and estimated their spirometry use both categorically (infrequent, expected, or highly frequent user) and numerically as an open-ended response. CF experience was defined as the number of years worked within the CF service. Estimation accuracy was evaluated using mean bias and mean absolute error (MAE).

**Results:**

Of the 29 HCPs invited to participate, 28 (96.6%) responded to the survey, including 10 (35.7%) physicians, 6 (21.4%) nurses, and 12 (42.9%) physiotherapists. There were 790 completed categorical estimates and 794 numerical estimates. The mean CF experience was 15.7 (SD 8.2) years. Across all roles, HCPs systematically underestimated home spirometry use (mean bias −4.9, SD 8.9; MAE 6.32, SD 8.1). No substantial differences in estimation accuracy were observed based on professional role, reported familiarity, or CF experience. Estimation accuracy declined with increasing actual spirometry use, with a strong negative Spearman rank correlation (ρ=−0.88; *P*<.001). HCPs tended to cluster their estimates within a narrow range, with a median of 4 (IQR 1-5) spirometry sessions per year.

**Conclusions:**

This study suggests that clinical perception by HCPs alone may not accurately reflect real-world engagement with home spirometry in people with CF. As CF care increasingly incorporates remote monitoring and virtual consultations, understanding actual patient engagement becomes increasingly important. Further qualitative and mixed methods research is needed to better understand the factors influencing home spirometry use and how different patterns of engagement can be effectively identified and supported.

## Introduction

The landscape of cystic fibrosis (CF) care has changed dramatically with the adoption of remote monitoring. The COVID-19 pandemic accelerated the integration of virtual clinics and remote monitoring into CF care. While the number of virtual clinic appointments has gradually declined since the peak in 2020, they still accounted for approximately 25% of outpatient consultations in adult centers in 2023, according to the Cystic Fibrosis Foundation’s annual report in the United States [1]. The Australian standards of care for people with CF clearly advocate telehealth, stating: “Standard 3: CF centres should have access to telehealth facilities” [2]. From the perspective of people with CF, a UK patient survey found that 62% of respondents preferred to include virtual clinics in their care, either primarily or in combination with in-person visits (hybrid care model) [3]. For telehealth to be effective, adequate access to remote monitoring is essential. Both the European Cystic Fibrosis Society and the UK standards of care listed several remote monitoring modalities, including but not limited to home spirometry, home sputum collection, pulse oximetry, and glucose monitoring [4,5]. Among various remote monitoring tools, both people with CF and CF health care professionals (HCPs) have consistently rated home spirometry as the most valuable [6]. A UK survey showed that home spirometry was widely available; 86.6% of the survey respondents used some form of remote monitoring, and 91.8% had access to home spirometry [3,7]. In addition, the UK standards of care and the US position papers endorsed home spirometry as a key component of virtual clinics and its use as an adjunct between clinic visits [5]. A UK study showed that home monitoring in CF could cost the government £1500 less per patient than routine in-person care [8]. Although the cost difference in this study was not statistically significant, the findings may encourage CF teams to reconsider resource allocation within their centers. Although questions remain about the interchangeability of home and clinic spirometry, emerging evidence supports the use of home spirometry in adults with CF. Unsupervised home spirometry has been shown to be comparable with clinic spirometry in terms of forced expiratory volume in the first second of expiration (FEV_1_) results, variability in home spirometry readings, and the spirometry quality based on the American Thoracic Society and European Respiratory Society grading [9-11].

Despite its growing popularity, the role of home spirometry in CF care presents several challenges, particularly regarding adherence to disease monitoring. Studies have shown different adherence rates: 1 study reported that 59% of people with CF completed weekly spirometry over a 1-year period, while another found that 60% to 83% of people with CF were adherent to 3-monthly spirometry (mean follow-up of 6.8 months) [12,13]. However, these were prospective studies and may reflect a more motivated or engaged group, limiting their generalizability to real-world settings. A recent observational Dutch study showed that 54% of people with CF stopped using their home spirometry device after the first year [14]. Similarly, a multicenter real-world pediatric study found that only 31% of children submitted at least 1 spirometry reading over a 20-month observation period [15]. As CF is a lifelong condition, studies often recruit both pediatric and adult populations as their participants. Some studies suggest that adults demonstrate poorer adherence or lower home spirometry uptake than adolescents and children in their post hoc analyses [14,16], whereas others showed no significant differences [17]. Relatively few studies have focused exclusively on home spirometry adherence in adults with CF, quoting a 72% adherence rate [9]. Despite variable adherence, evidence from multiple studies supports the benefits of home spirometry. The implementation of home spirometry was associated with the detection of more pulmonary exacerbations and improvements in Cystic Fibrosis Questionnaire-Revised scores, a validated health-related quality of life score used in people with CF [8,9]. Home spirometry also influences clinical decision-making. A recent study by Tan et al [15] showed that 72% of CF clinicians reported that home spirometry affected their clinical decisions, and its use reduced the need for people with CF to return to clinic for follow-up spirometry [9]. Therefore, the benefits of home spirometry are dependent on sustained patient engagement, as infrequent testing may reduce its value for remote monitoring, early detection of pulmonary exacerbations, and treatment decision-making.

A study exploring clinicians’ perspectives on remote monitoring reported variation in engagement among people with CF, ranging from individuals who engaged consistently to those who disengaged over time because of psychosocial barriers or failed to engage altogether [6]. As clinicians increasingly rely on home spirometry within hybrid models of care, an accurate understanding of patient engagement becomes important. Inaccurate assumptions regarding monitoring behaviors may influence resource allocation, interpretation of remote monitoring data, and clinical decision-making. Little is known about whether HCPs can accurately estimate how people with CF engage with home spirometry in real-world settings.

In this study, we aimed to evaluate HCPs’ ability to estimate home spirometry use among adults with CF in 2024 and compare these predictions with recorded spirometry use data. We also explored whether estimation accuracy varied by HCP role (physicians vs nurses vs physiotherapists), self-reported familiarity with each individual with CF, and the extent of their CF experience, defined as the number of years working within the CF center.

## Methods

We conducted a cross-sectional study at a single adult CF center. Survey dissemination and collection were conducted in January 2025, using spirometry records from January to December 2024.

### Spirometry Data

Home spirometry was implemented at our center in 2020. Spirometry data were extracted from the AirNext portable home spirometer (NuvoAir). As per our local clinical care pathway, we routinely review people with CF at least every 3 months, and they are expected to perform spirometry prior to clinic review and during periods of increasing clinical symptoms. Hence, we expect them to perform around 4 to 6 spirometry sessions per year. The home spirometry system comprises 3 components: the handheld AirNext device, a patient-facing smartphone app, and a clinician portal that provides centralized access to spirometry data. The device connects to the patient’s smartphone via Bluetooth, with all spirometry data automatically uploaded to both the app and clinician portal.

All people with CF received initial training during rollout and were instructed to perform at least 3 maneuvers per session. The NuvoAir algorithm selects the highest-quality reading in accordance with American Thoracic Society and European Respiratory Society guidelines and uploads this result to the platform [18]. Patients are expected to perform spirometry prior to all clinic visits (virtual or in-person). If unavailable, spirometry is either performed during the clinic or requested afterward. However, no formal follow-up is undertaken unless there is clinical concern. Only spirometry sessions conducted in 2024 were included, either performed at home or as part of routine clinic reviews. Sessions performed in the context of clinical trials, nebulized antibiotic or mucolytic challenges were excluded. Serial spirometry sessions performed during inpatient admissions, home intravenous antibiotic therapy, or both, were counted as a single encounter to prevent possible bias due to an awareness of individuals who more frequently exacerbate. Hospital records for all people with CF included in the study were cross-referenced with spirometry records to ensure these criteria were met.

### Survey Design

#### Overview

A paper-based survey was developed to capture HCPs’ retrospective estimates of spirometry behavior in people with CF for the year 2024. The survey was not externally validated but underwent internal review by the authors. Survey dissemination and data collection were completed in January 2025. CF care is inherently multidisciplinary, reflecting the multisystem nature of the disease. In the United Kingdom, the multidisciplinary team typically includes physicians, CF specialist nurses, physiotherapists, dietitians, clinical psychologists, and social workers—with every person with CF having access to these team members at every clinic review. For the purposes of this study, only selected multidisciplinary team members were considered: physicians, CF specialist nurses, and physiotherapists who had direct clinical involvement with people with CF and routine exposure to home spirometry. All HCPs were permanent staff—no rotational staff were included in this study. In the United Kingdom, dedicated respiratory therapists are not routinely part of the health care workforce. Instead, physiotherapists fulfill a similar role in respiratory care. At our center, physicians and physiotherapists are trained to perform home spirometry and coach people with CF in its use. Some CF specialist nurses are also trained to coach spirometry. As they are often the first point of contact for people with CF, all CF specialist nurses are trained in spirometry interpretation and the clinical significance of lung function in CF care. The center provides care for 476 people with CF aged 17 years or older. People with CF who had lung transplants were excluded. Of these, 63% (300/476) people with CF had spirometry records in predefined use categories in 2024 (n=73, 24.3% people with CF with 0‐1 sessions/year; n=127, 42.3% with 4‐6 sessions/year; and n=100, 33.3% with ≥10 sessions/year). From each category, 16 people with CF were randomly selected, resulting in a total sample of 48 people with CF. This sample size was chosen pragmatically to ensure the survey was feasible to complete while maintaining sufficient representation for a meaningful analysis. The predefined use categories were infrequent users (0‐1 sessions/year), expected users (4‐6 sessions/year), and highly frequent users (≥10 sessions/year).

The names of people with CF were presented to HCPs during the survey to enable clinical recall. Actual spirometry counts were blinded during the survey. After data collection, names were redacted and replaced with research numbers. An anonymized version of the survey is provided in Multimedia Appendix 1. The survey consisted of 3 sequential parts.

#### Familiarity Assessment

HCPs were first asked to indicate their familiarity with each patient using a 3-point Likert scale:

- Level 1: “I do not know the patient at all”
- Level 2: “I know the patient somewhat (limited clinical recall)”
- Level 3: “I know the patient well (clear understanding of their clinical background)”

Only HCPs who answered level 2 and level 3 for a patient could proceed to answer the questions on spirometry estimation below.

#### Categorical Estimation

HCPs were asked to estimate the total number of spirometry sessions for each person with CF during 2024, selecting from one of three categories (1) “rarely performs spirometry (0‐1 sessions/year),” (2) “around expected frequency (4‐6 sessions/year),” and (3) “more than expected (≥10 sessions/year).”

These categories corresponded to the predefined use groups used for patient selection. The rationale for the categorical estimation was to evaluate the HCPs’ ability to correctly identify which people with CF were rare, expected, or frequent spirometry users. Therefore, we deliberately created spirometry frequency groups with clear distinctions between them.

#### Numeric Estimation

HCPs were then asked to provide a discrete numeric estimate of the number of spirometry sessions each person with CF performed in 2024. This complemented the categorical estimates and added greater granularity to the data. Unlike the categorical estimates, we did not assess accuracy based on an exact match with the true value, as it was unrealistic to expect HCPs to identify the precise number of sessions. Instead, we evaluated the degree of bias and mean absolute error (MAE), which we considered more pragmatic and informative.

### Statistical Analysis

All statistical analyses and visualizations were performed using RStudio (version 2025.05.0+496; Posit PBC). Bias was defined as the difference between the estimated and actual number of spirometry sessions (estimate−actual). A positive bias was reported as “overestimate,” that is, the estimated number of spirometry sessions was higher than the actual number; a zero bias was reported as “correct,” that is, the estimated number of spirometry sessions equaled the actual number; and a negative bias was reported as “underestimate,” that is, the estimated number of spirometry sessions was less than the actual number. While bias indicates the direction (negative or positive), MAE indicates the precision of the estimates. MAE is defined as the average of the absolute differences between estimates and actual values. Comparative analyses were conducted by HCP role (physicians, nurses, and physiotherapists) and familiarity level (level 2 vs level 3). Statistical tests included the chi-square test of independence (HCP role vs categorical estimation of spirometry use), Wilcoxon rank-sum test (level of familiarity vs mean bias or MAE), Kruskal-Wallis test (HCP role vs mean bias or MAE), and Spearman rank correlation (numerical estimation of spirometry use vs actual spirometry use and CF years of experience vs mean bias or MAE). No weighting was applied to account for differences in the numbers of respondents across HCP groups, as between-group comparisons were based on standardized metrics (proportions, mean bias, and MAE) that inherently account for differences in group size.

### Ethical Considerations

Ethics approval was obtained from the North West–Greater Manchester East Research Ethics Committee (IRAS 340274). The survey participants were health care providers from our CF center, and participation in the study was voluntary, with no compensation provided. Participants were informed that completion and return of the paper survey would be taken as implied consent to participate, while nonreturn of the survey was considered a decision not to participate. Apart from their clinical role, no other identifiable data were collected from the participants. Regarding patient data, this was a noninterventional study, and the authors were part of the direct clinical care team for the patients. Therefore, additional patient consent was not sought. All patient data were obtained from routine clinical records and pseudonymized prior to analysis.

## Results

### Overview

The survey achieved a response rate of 96.6%, including 28 of the 29 HCPs invited to participate. Of the 28 respondents, 10 (100%) were physicians, 6 (85.7%) were nurses, and 12 (100%) were physiotherapists. Analyses were restricted to responses where participants rated their familiarity with the patient as level 2 or level 3, indicating sufficient clinical knowledge to recall patient history. Physicians reported familiarity with 16.7% to 100% of patients, nurses with 12.5% to 77.1%, and physiotherapists with 10.4% to 79.2%. All 48 patients were familiar to at least 1 HCP.

### Categorical Estimation

**Figure 1.**
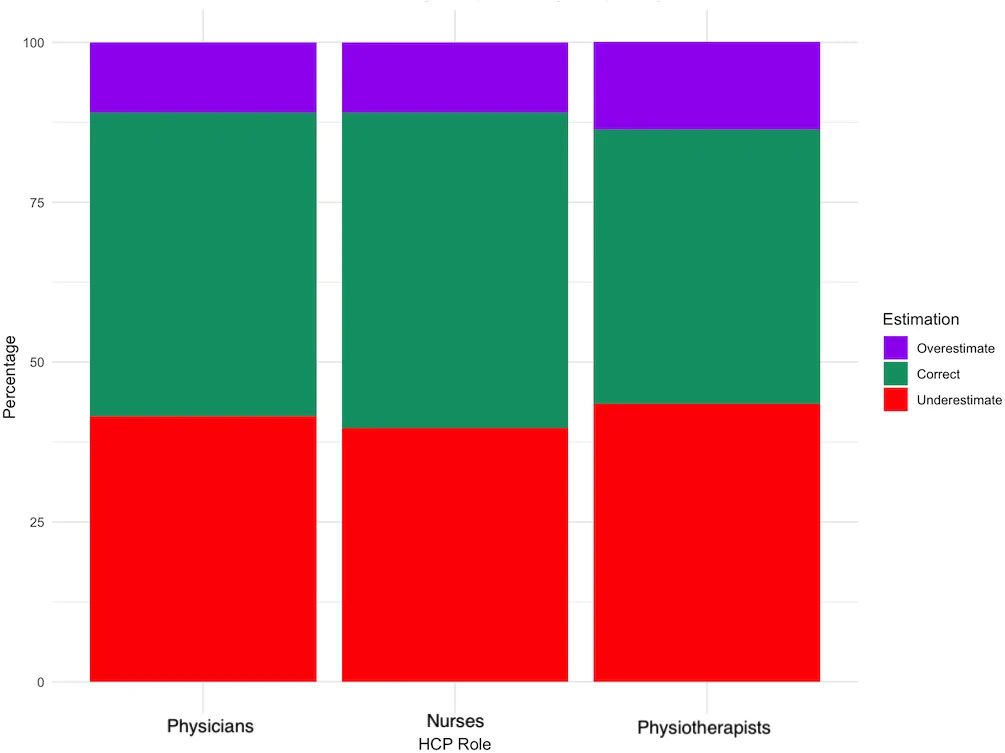
Stacked bar charts showing the percentage distribution of health care professionals’ (HCPs’) estimation of actual spirometry use (correct estimation, overestimation, and underestimation), grouped by HCP role (physicians, nurses, and physiotherapists).

A total of 790 categorical responses were analyzed. HCPs frequently incorrectly estimated spirometry use among people with CF: 332 (42%) responses underestimated use, 96 (12.2%) responses overestimated, and 362 (45.8%) responses were correct. HCPs who rated themselves as “very familiar” with the individual (level 3) were no more likely to estimate correctly than those who were “somewhat familiar” (level 2; *P*=.77). There was also little difference in accuracy between the different HCP roles, as shown in Figure 1. Nurses had the highest rate of correct estimation (67/136, 49.3%), while physiotherapists had the lowest (144/336, 42.9%), along with the highest rates of both underestimation (146/336, 43.5%) and overestimation (46/336, 13.7%). There was no statistically significant association between HCP role and direction of estimation (*P*=.61). Table 1 shows the detailed breakdown by use group (infrequent, expected, or highly frequent users) against the estimation accuracy (underestimation, correct estimation, and overestimation).

**Table 1.** Health care professionals’ estimation of spirometry use among people with cystic fibrosis.

Spirometry use	Correct estimation, n (%)	Overestimation, n (%)	Underestimation, n (%)
Infrequent users (0‐1 sessions/year; n=221)	149 (67.4)	72 (32.6)	0 (0)
Expected users (4‐6 sessions/year; n=287)	157 (54.7)	24 (8.4)	106 (36.9)
Highly frequent users (≥10 sessions/year; n=282)	56 (19.9)	0 (0)	226 (80.1)

### Numerical Estimation

**Figure 2.**
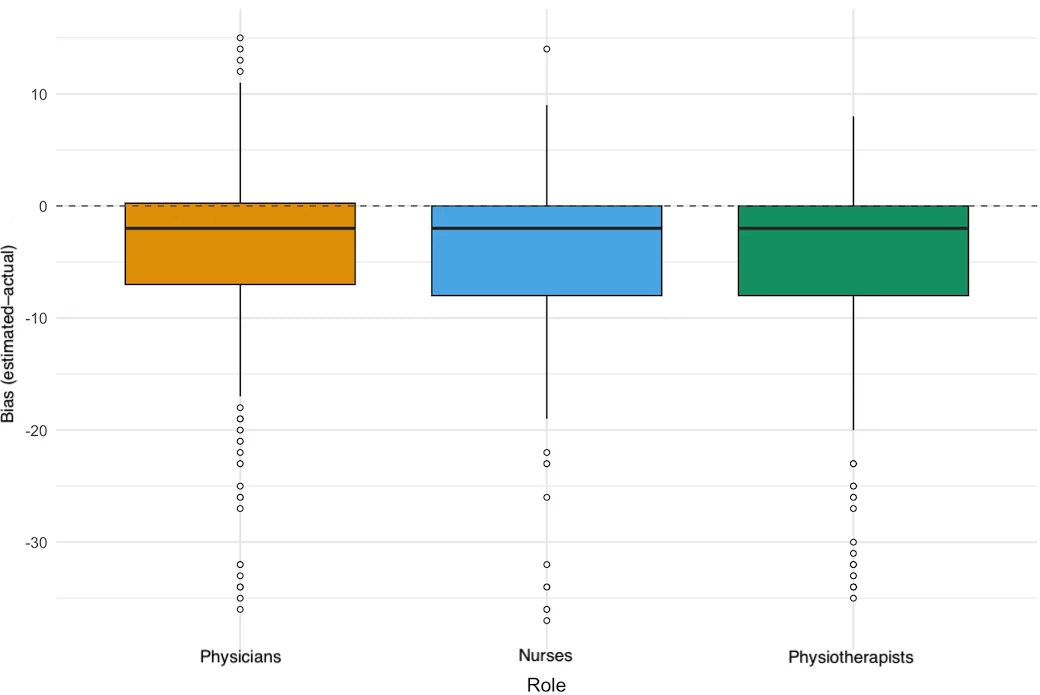
Boxplot showing the bias (estimated spirometry use minus actual spirometry use) across the different health care professional (HCP) roles. A positive bias value indicates overestimation, while a negative bias value indicates underestimation.

**Figure 3.**
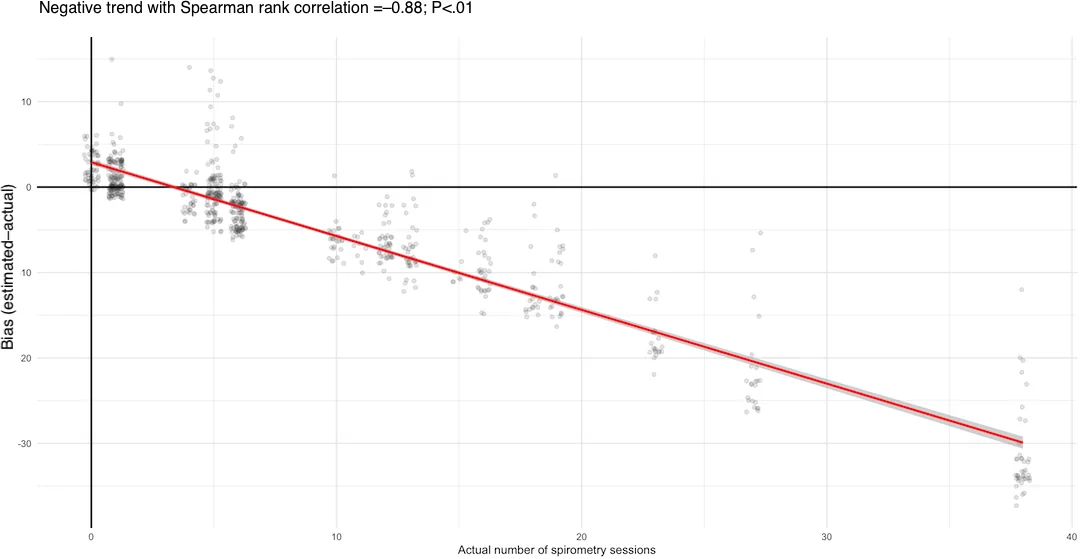
Relationship between the actual number of spirometry sessions and the estimated error or bias. Each gray dot represents 1 response (ie, there are 794 dots on the graph). The red line indicates the trend in the data.

**Figure 4.**
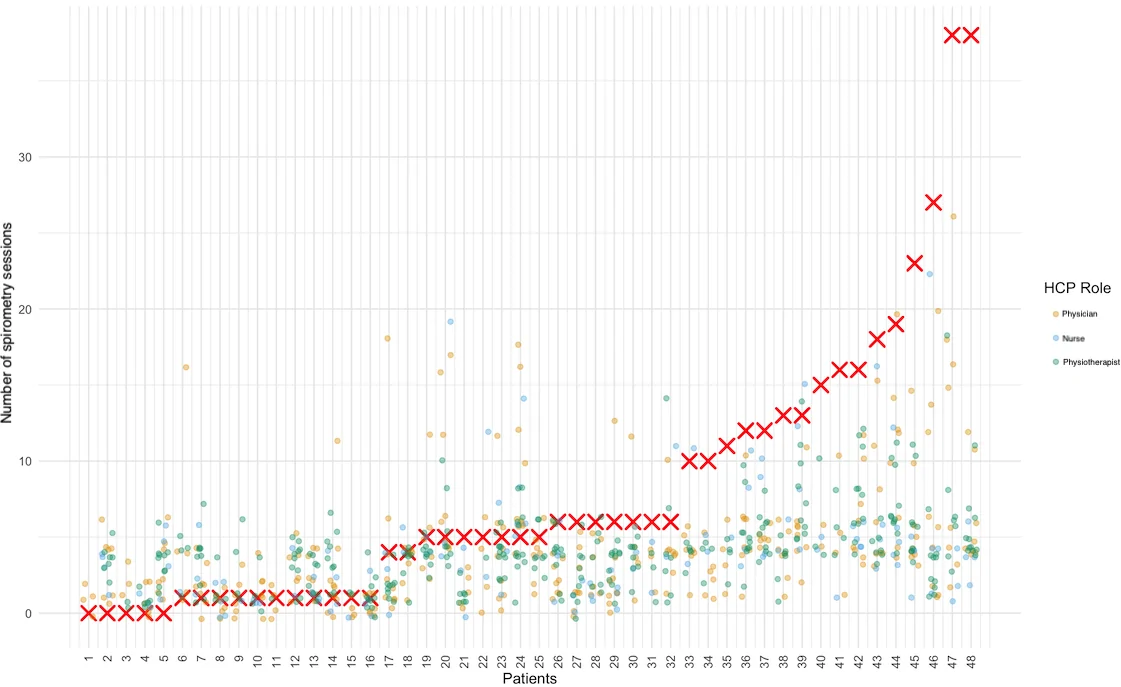
Actual and estimated numbers of spirometry sessions for individual patients. The x-axis represents individual patients (patients 1-48). The red crosses represent the actual number of spirometry sessions performed by each patient in 2024. The dots represent the estimated number of spirometry sessions by the health care professionals (HCPs; yellow for physicians, blue for nurses, and green for physiotherapists).

There were 794 completed responses for the numerical estimation of spirometry frequency. We assessed accuracy using mean bias and MAE. Overall, the mean bias was −4.91 (SD 8.9) and the MAE was 6.32 (SD 8.1), indicating a consistent trend toward underestimation and moderate variability in responses. Figure 2 shows a boxplot comparing the mean biases across HCP roles, ranging from −4.50 (SD 8.6) to −5.13 (SD 8.9). MAEs were similar across roles, ranging from 6.13 (SD 7.5) to 6.47 (SD 8.6). This suggested that all HCP roles underestimated spirometry use with similar imprecision, with no significant differences between roles (*P*=.86 for mean bias; *P*=.78 for MAE). We observed that the numerical estimation accuracy (defined by bias rather than exact estimate) declined with increasing actual spirometry use, with a strong negative Spearman rank correlation (ρ=−0.88; *P*<.001; Figure 3). Additionally, HCPs tended to cluster their estimates within a narrow range, with a median of 4 (IQR 1-5) spirometry sessions per year across all people with CF, and similar medians were observed across all HCP roles (Figure 4). Similar to categorical estimation, there were no significant differences in bias or MAE by familiarity level (*P*=.89 and *P*=.30, respectively).

### Years of Experience

The mean duration of HCPs’ CF experience, expressed as years of working at the center, was 15.7 (SD 8.2) years, ranging from 2 to 31 years (mean 14.8, SD 7.8 years among physicians, mean 17.9, SD 7.2 years among nurses, and mean 15.7, SD 8.7 years among physiotherapists). There was no significant association between years of CF experience and mean bias or MAE (*P*=.58 and *P*=.81, respectively).

## Discussion

### Principal Findings

Our findings demonstrate that CF HCPs were generally poor at accurately predicting home spirometry use among people with CF. First, our study highlights a tendency to underestimate the number of spirometry sessions performed by people with CF attending the center. This underestimation occurred across all roles and regardless of HCPs’ self-reported familiarity with people with CF attending the center. Second, CF experience, defined as the number of years an HCP has worked at the CF center, did not show any strong signal in predicting home spirometry use. Both findings suggest that perceived clinical insight may not reliably translate to accurate evaluation of remote monitoring behavior. This is an important observation, particularly as home monitoring becomes more integrated into clinical care and models of care continue to evolve.

To our knowledge, no previous study has evaluated whether CF HCPs can accurately estimate people with CF’s engagement with home spirometry using objective data. Previous studies involving clinician-reported estimates have primarily focused on treatment adherence rather than disease monitoring. For example, Daniels et al [19] reported that HCPs tended to overestimate adherence to nebulized therapy when compared with electronic monitoring data. Although home spirometry and nebulized treatment represent distinct health behaviors, both are performed unsupervised at home and depend on patient-initiated engagement. It is therefore interesting that previous work reported clinician overestimation of nebulized treatment adherence, whereas our study found underestimation of home spirometry use. This contrast should be interpreted cautiously, but it suggests that clinicians may perceive treatment adherence and disease monitoring differently.

In our center, we specifically evaluated CF HCPs who were directly involved in home spirometry, namely physicians, nurses, and physiotherapists. This role may differ from other centers. While we acknowledge that the nature and extent of input on home spirometry may vary among HCPs, it is important to note that all HCPs in our study showed a similar directional pattern of underestimation in their predictions. This consistency highlights the need to improve understanding of home spirometry across all professional roles.

Our findings suggest that HCPs are better at identifying people with CF who underuse home spirometry than those who overuse it. Figure 3 shows the inverse relationship between the actual number of spirometry sessions and estimation accuracy. The more frequently an individual performed spirometry, the more likely CF caregivers were to underestimate it. While recognizing people with CF who are not engaging with home spirometry is important, it is equally crucial to identify those who tend to use it more frequently and to understand the underlying drivers of this behavior. Anxiety around home spirometry may be a contributing factor. In a survey of 90 people with CF, 83% reported experiencing at least mild anxiety related to home spirometry [20]. It is possible that HCPs’ perceptions of spirometry as an anxiety-inducing procedure contribute to their tendency to underestimate use among people with CF, although this remains speculative. Interpretation of home spirometry during periods of clinical stability can also be challenging, as lung function may vary substantially (around 15% FEV_1_) without leading to pulmonary exacerbation [21]. This creates uncertainty for some people with CF about how to interpret and respond to home spirometry results. In a mixed methods study of home monitoring in adults with CF in the United Kingdom, although many participants found monitoring reassuring and empowering, a minority found frequent monitoring more challenging, describing feeling “a bit more anxious or a bit more worried,” “a bit more paranoid,” or that spirometry became “like an obsession that you want to know all the time” [8]. These observations suggest that high-frequency home spirometry use should not simply be viewed as “good adherence” but may represent different monitoring phenotypes. This concept of different monitoring phenotypes is supported by a mixed methods study by Oppelaar et al [6], where they described several patterns of engagement with remote monitoring: people with CF who never initiated monitoring, those who discontinued because of technical or psychosocial barriers, those who used monitoring only when clinically indicated, and those who engaged regularly with the program. We similarly observed marked heterogeneity in spirometry behaviors within our cohort despite protocolized recommendations at our center. The rationale for use may be a contributing factor to the observed heterogeneity. Tan et al [15] found that 48% of children with CF performed home spirometry as part of routine follow-up, whereas 33% performed spirometry in response to illness-related telephone contact with their CF team.

Exploring the reasons for high-frequency spirometry use was not part of the aim of this study. This may reflect proactive self-management or fluctuating respiratory symptoms, which carry a psychological burden associated with symptom monitoring. Understanding these factors is important to mitigate potential unintended risks of spirometry monitoring. Further mixed methods or qualitative research is needed to explore these behaviors in greater detail.

We recognize several limitations in this study. The survey design may have inadvertently introduced anchoring bias. Participants were asked to provide both a categorical and a numerical estimate of spirometry use on the same form. This may have led some HCPs to default to the values provided in the survey (ie, 0, 1, 4, 6, or 10). Furthermore, the clustering of estimates around 4 sessions per year, with a narrow IQR of 1 to 5, suggests a central tendency bias, where HCPs tended to avoid selecting extreme values. This pattern may reflect normative expectations overriding clinical recall, indicating that estimates may be influenced more by perceived care-pathway policy than by recollection of individual patient behavior. The categorical question presented HCPs with 3 options: infrequent users (0‐1 sessions/year), expected users (4‐6 sessions/year), and highly frequent users (≥10 sessions/year). By design, this limited their ability to underestimate infrequent users or overestimate highly frequent users. To address this, we included an open-ended numerical estimation section, allowing HCPs to provide a more nuanced answer regarding the spirometry frequency estimates. Grouping patients into 3 categories (infrequent, expected, and highly frequent users) was a deliberate simplification to facilitate clinical recall and does not reflect the true distribution of spirometry use within our cohort. For context, 15.6% (73/469) of patients completed 0‐1 sessions, 27.1% (127/469) completed 4‐6 sessions, and 21.3% (100/469) completed ≥10 sessions in 2024 (these figures represent raw spirometry frequency and were not adjusted to our study definition of a single spirometry session—refer to the Methods section). This sampling strategy may overrepresent behavioral extremes and exaggerate bias. Results should therefore be interpreted as HCPs’ ability to discriminate between predefined groups rather than as estimates of population-level predictive accuracy. Clinical recall and familiarity with an individual with CF are inherently subjective and can vary significantly. An HCP may be familiar with certain aspects of their care but not with their home spirometry behavior. Additionally, people with CF at either end of the clinic attendance spectrum (those who attend very frequently or very rarely) may stand out more in an HCP’s memory, potentially influencing estimation accuracy. HCPs in our study also work across varied clinical settings, including outpatient, inpatient, and home-based care. HCP familiarity with a given individual is often shaped by their clinical needs. For example, those working primarily on the wards may be more familiar with people with CF requiring frequent admissions. To provide a fair representation of clinical familiarity, we included only people with CF for whom the HCP reported a familiarity score of 2 or higher on the survey’s familiarity scale. Finally, each HCP provided multiple estimates across patients, and these were analyzed independently. Our data were not adjusted to account for within-HCP clustering. The results should be interpreted as exploratory and descriptive.

### Future Directions

As CF care increasingly incorporates remote monitoring into everyday practice, it is essential that we understand how people with CF are actually using these tools. At its core, home spirometry promotes autonomy and self-directed care in people with CF, but it carries the risk of shifting the burden from the disease itself to the act of monitoring. Therefore, understanding individual behavior around remote monitoring and being able to identify both underusers and overusers, along with the triggers behind these patterns, are crucial. This study identified that HCPs’ estimation of home spirometry use was exceptionally poor in people with CF who undertook frequent spirometry, that is, more than expected as per our local standards. Exploring the reasons for high-frequency spirometry use was not part of the aim of this study. This may reflect proactive self-management or fluctuating respiratory symptoms, which carry a psychological burden associated with symptom monitoring. Understanding these factors is important to mitigate potential unintended risks of spirometry monitoring. Further mixed methods or qualitative research is needed to explore these behaviors in greater detail.

Errors in estimating spirometry frequency may have important clinical implications. Inaccurate assumptions about spirometry use could inadvertently affect prioritization of health care resources, introduce cognitive biases in the interpretation of health behaviors, and influence how clinical conversations are framed, as well as subsequent decisions regarding patient follow-up. Given the inaccuracy of clinical estimates, more robust system-wide approaches to presenting objective adherence data should be adopted, such as automated spirometry alerts integrated into health care records.

### Conclusions

This study highlights a systematic underestimation of spirometry use by CF HCPs in people with CF, regardless of their professional role, perceived familiarity with the individual, or level of CF experience (measured by years working at a CF center). Our findings suggest that clinical intuition alone may not reliably reflect how adults with CF engage with home spirometry in real-world settings. As hybrid models of care increasingly incorporate remote monitoring and virtual consultations, an accurate understanding of patient engagement with home spirometry becomes increasingly important. The discrepancy between HCPs’ perception and objectively recorded spirometry behavior highlights an important gap in our understanding of remote monitoring engagement. Further qualitative and mixed methods research involving both people with CF and HCPs is needed to better understand the motivations, barriers, and different phenotypes of home spirometry use and how these behaviors can be appropriately identified and supported.

## Supplementary material

10.2196/93545 Multimedia Appendix 1 Anonymized survey.

## Abbreviations

CF: cystic fibrosis
FEV_1_: forced expiratory volume in the first second of expiration
HCP: health care professional
MAE: mean absolute error
