# Supplementary material for: Health Care Professionals’ Estimation of Home Spirometry Use Among People With Cystic Fibrosis: Cross-Sectional Study
Source: JMIR Form Res. 2026 Aug 26;10:e93545. doi: 10.2196/93545 (PMC13513205; doi:10.2196/93545)
Supplement: Supplementary file 1 [file formative-v10-e93545-s001.docx]

**Perceived Patient Adherence to Home Spirometry by CF Healthcare Professionals**

This survey is designed to explore how well CF healthcare professionals know their patients and how accurately they perceive their adherence to home spirometry during the year 2024. We aim for a patient doing spirometry once every 3-months

For each patient listed below, please answer the following three questions:

1. **Indicate how familiar you are with the patient.**
2. **Estimate how frequently the patient has performed home spirometry in 2024 by selecting one of the given frequency categories.**
3. **Provide your best estimate of the exact number of home spirometry sessions the patient completed in 2024.**
   *Please provide a single number (e.g.,* **✓** 4, **✗** 4 to 5 **✗** 4 - 5)

**Important:**
If you do **not** know the patient at all, please **skip questions 2 and 3**.

## 1. Familiarity Scale

1 – I do not know the patient at all

2 – I know the patient a bit (limited clinical recall)

3 – I know the patient well (clear understanding of their clinical background)

## 2. Estimated Home Spirometry Frequency in 2024

☐ Rarely performs spirometry (0–1 sessions/year)

☐ Around expected frequency (4–6 sessions/year)

☐ More than expected (≥10 sessions/year)

3. **Please also write the approximate number of sessions you think this patient performed in 2024.**

**Please note:**

- Spirometry for clinical trials, inhaled antibiotic challenges or during oral antibiotics treatment for CF exacerbation **should NOT be counted.**

- Spirometry performed during inpatient IV, home IV or inpatient with subsequent home IV or oral continuation, should be counted as **one spirometry session.**

# Survey Responses

| Patient Name | Familiarity (1-3) | Tick Frequency Category (Only if Familiarity is 2 or 3) | Estimated Number of Sessions in 2024 (Only if Familiarity is 2 or 3) |
| --- | --- | --- | --- |
| Patient 1 | ☐ 1 ☐ 2 ☐ 3 | ☐ Rarely (0–1) ☐ Expected (4–6) ☐ More than expected (≥10) |  |
| Patient 2 | ☐ 1 ☐ 2 ☐ 3 | ☐ Rarely (0–1) ☐ Expected (4–6) ☐ More than expected (≥10) |  |
| Patient 3 | ☐ 1 ☐ 2 ☐ 3 | ☐ Rarely (0–1) ☐ Expected (4–6) ☐ More than expected (≥10) |  |
| Patient 4 | ☐ 1 ☐ 2 ☐ 3 | ☐ Rarely (0–1) ☐ Expected (4–6) ☐ More than expected (≥10) |  |
| Patient 5 | ☐ 1 ☐ 2 ☐ 3 | ☐ Rarely (0–1) ☐ Expected (4–6) ☐ More than expected (≥10) |  |
| Patient 6 | ☐ 1 ☐ 2 ☐ 3 | ☐ Rarely (0–1) ☐ Expected (4–6) ☐ More than expected (≥10) |  |
| Patient 7 | ☐ 1 ☐ 2 ☐ 3 | ☐ Rarely (0–1) ☐ Expected (4–6) ☐ More than expected (≥10) |  |
| Patient 8 | ☐ 1 ☐ 2 ☐ 3 | ☐ Rarely (0–1) ☐ Expected (4–6) ☐ More than expected (≥10) |  |
| Patient 9 | ☐ 1 ☐ 2 ☐ 3 | ☐ Rarely (0–1) ☐ Expected (4–6) ☐ More than expected (≥10) |  |
